# Significance of Preoperative Multidisciplinary Assessment with 30-Second Sit-to-Stand and Timed Up-and-Go Tests in Predicting Postoperative Outcomes

**DOI:** 10.3390/jcm14041085

**Published:** 2025-02-08

**Authors:** Mücahid Osman Yücel, Sönmez Sağlam, Raşit Emin Dalaslan, Mehmet Arıcan, Zekeriya Okan Karaduman, Bedrettin Akar, Mücahit Çelik, İsmail Sav

**Affiliations:** 1 Department of Orthopaedics and Traumatology, Faculty of Medicine, Duzce University, 81620 Duzce, Türkiyerasitdalaslan@duzce.edu.tr (R.E.D.);; 2 Department of Orthopedics and Traumatology, Sakarya Yenikent State Hospital, 54290 Sakarya, Türkiye; 3 Department of Orthopaedics and Traumatology, Faculty of Medicine, Binali Yıldırım University, 24002 Erzincan, Türkiye

**Keywords:** knee prosthetic arthroplasty, functional recovery, ASA physical status classification, postoperative period

## Abstract

**Background/Objectives:** Evaluating basic daily activities like sitting, standing, and walking is crucial for predicting preoperative risks and postoperative recovery. These functional abilities can be assessed through patient history or measured using objective tests. For this purpose, the 30-Second Sit-to-Stand (30STS) Test and Timed Up-and-Go (TUG) Test are frequently used in clinical settings. However, few studies have evaluated their effectiveness in anesthesia and orthopedics. In this study, we aimed to assess the applicability of these tests across clinical disciplines. **Methods:** A total of 43 patients who underwent total knee arthroplasty (TKA) surgery between January and December 2023 with American Society of Anesthesiologists (ASA) scores of 2–3 were retrospectively evaluated. The 30STS, TUG, and VAS scores were recorded preoperatively and on postoperative days 90–180. **Results:** The preoperative 30STS and TUG scores showed no statistically significant difference between the ASA 2–3 groups, but the ASA 2 group demonstrated a more pronounced performance improvement in both tests during the first 90 days postoperatively. The correlation tests revealed a strong positive relationship with the TUG Test and a moderate positive relationship with the 30STS and VAS scores. **Conclusions:** The correlation between the preoperative and postoperative results of the 30STS and TUG Tests suggests that preoperative tests can predict post-operative functional performance. However, the lack of a significant statistical relationship between the preoperative tests and ASA scores indicates that these tests may not be sufficiently useful for assessing the functional capacity. The better test outcomes in the ASA 2 patients indicate that combining these assessments with anesthetic evaluations may improve postoperative functional predictions, thereby promoting a multidisciplinary approach.

## 1. Introduction

The two most important postoperative changes observed in patients undergoing TKA are reduction in pain and improvement in walking ability [1]. A proportion of 96.2% of patients think that their walking ability will be better after surgery [2]. Basic movements such as walking short distances and getting up from a chair are critical for independent living [3]. Therefore, specialized tests that measure the functional performance of patients are of great importance to evaluate patients’ capacity to lead an independent life and the recovery process both preoperatively and postoperatively.

Measurement devices such as electromyography, force platforms, and optokinematic systems are used to evaluate the functional performance of patients; however, these methods are time-consuming and require sophisticated laboratories [4,5]. Therefore, simple and rapidly applicable tests may be preferred in clinical practice. The 30STS and TUG Tests are among such simple and rapid tests. The 30STS Test evaluates physical performance by measuring how many times a person can sit and stand in 30 s [6]. The TUG Test, on the other hand, assesses a patient’s ability to sit up and walk with a simple time measurement [7]. Both tests are easy-to-administer methods that do not require equipment and are frequently used to evaluate movements that are important in daily life, such as walking and sitting up. The measurement of functional performance with these tests in patients with TKA and evaluation of their relationship with ASA scores may contribute to the use of these tests for different purposes by many clinical branches such as physical therapy, orthopedics, and anesthesia [7,8].

The ASA (American Society of Anesthesiologists) classification is a system used to assess the preoperative health status of patients and to determine the risks that may be encountered during surgery. This classification creates a risk profile by taking into account the general health status and functional limitations of the patients. In the ASA classification, the patient’s ability to perform physical activities plays an important role. Patients in ASA 2 class are not severely affected in their activities of daily living despite having mild health problems, whereas patients in ASA 3 class may have significant difficulties in stair climbing or light physical activities due to more serious diseases [8]. Functional limitations are an important part of this classification because the extent to which the patient can perform daily activities provides clues about the postoperative recovery process and complication risks [9,10]. Therefore, the ASA classification is widely used to understand the patient’s general health status and the risks associated with surgery before surgical interventions [11].

Before TKA, patients are evaluated in terms of functional abilities not only by orthopedists and physiotherapists but also by anesthesiologists. Orthopedists need the ASA classification when making surgical decisions and anesthesiologists need ASA classification to determine the patient’s risk of complications. During ASA scoring, anamnesis is taken from the patients and their performance in performing daily tasks is evaluated [11]. In the literature, there are studies showing that tests showing functional abilities such as the 6-minute walk test are compatible with ASA scores [12]. However, there is no study examining the concordance of the more practical 30STS and TUG Tests with ASA scores. In this study, we aimed to examine the correlation of the preoperative and postoperative results of the 30STS and TUG Tests with ASA values and the relationship between these results and the patients’ Visual Analog Scale (VAS) scores.

## 2. Materials and Methods

This retrospective observational study was conducted on patients who underwent TKA surgery between January 2023 and December 2023. The aim of the study was to investigate the effects of 30STS and TUG Tests on the functional performance and ASA scores of patients in the preoperative and postoperative period. The necessary approval was obtained from an ethics committee (Ethics Committee Approval No: date: 21 October 2024; registration number: 2024/216) and permission was obtained from the patients by signing a written consent form.

All patients were treated in the same surgical center by the same surgical and anesthesia teams within the framework of standard protocols. All patients underwent a posterior stabilized TKA through a median parapatellar incision under regional anesthesia and tourniquet guidance. In addition, standard pain management and physical therapy protocols were applied for all patients in the postoperative period.

Patients who underwent TKA surgery underwent a multidisciplinary rehabilitation program from the first postoperative day. Within the scope of this protocol, patients were mobilized within 24 h postoperatively, and in the early postoperative period, range of motion was appropriately increased with active exercises and the CPM (continuous passive motion) device (Artromot-K1 Comfort, Ormed GmbH, Freiburg, Germany). The main goal of rehabilitation was to control pain, increase joint mobility, and reduce the risk of complications. Patients also performed quadriceps strengthening exercises, isometric exercises, and knee flexion and extension exercises in the postoperative period. All applications were performed regularly by experienced physiotherapists during hospitalization in the ward and the patients were given the necessary training to continue this protocol recommended by the TOTBID (Turkish Orthopaedic and Traumatology Association) at home [13].

The data of all patients were recorded preoperatively and postoperatively by the same physiotherapist. This physiotherapist did not have any information other than the demographic data of the patients, thus maintaining the objectivity and impartiality of the study. Physical performance assessments were performed by a single specialist to ensure consistency of clinical results.

A total of 43 patients aged between 60 and 81 years, who underwent TKA surgery between January 2023 and December 2023, were included in the study. Preoperative demographic characteristics and ASA values were recorded. Inclusion criteria were patients who regularly attended outpatient clinic visits, had sufficient clinical data, and had ASA scores of 2–3. Exclusion criteria were as follows: patients who had undergone any other surgical intervention other than knee replacement, patients with a history of medical procedures or diagnoses that could affect the results during follow-up, and patients with missing preoperative or postoperative follow-up data. As the study is retrospective, no prior sample size calculation was performed, and all eligible patients were included in the study.

The 30-Second Sit-to-Stand Test (30STS): In this test, patients were asked to sit up and sit down as many times as possible within 30 s from a standard chair 43 cm high, crossing their arms over their chest. The test was performed by an experienced physiotherapist in a quiet and brightly lit room. The aim of the test was to assess lower limb muscle strength and functional capacity. The validity and reliability of the 30STS test have been confirmed in previous studies [6]. The test was administered in the preoperative period and on postoperative days 90 and 180.

The Timed Up-and-Go Test (TUG): In this test, patients were asked to get up from the same chair, walk a distance of 3 m marked on the floor at a normal speed, return and sit back on the chair. The duration of the test was measured with a stopwatch from the beginning of the movement to sitting down again. The TUG Test was performed by the same physiotherapist on a quiet and flat surface. The validity and reliability of the test in assessing functional mobility and balance have been proven in the literature [7]. The application was performed in the preoperative period and on postoperative days 90 and 180. The mean values for the 30STS and TUG Tests according to age and gender can be found in Appendix A, Table A1 [14,15].

ASA Classification: The ASA scores of the patients were determined by an experienced anesthesia team during preoperative evaluation. Patients’ current medical conditions, systemic diseases, and ability to perform physical activities were taken into account during the evaluation process. It is known that ASA classification can be subjective and so standardization of assessments is important; therefore, all assessments were performed by the same anesthesia team following a standard protocol. According to ASA scoring, patients with comorbidities that were under control and did not limit daily activities were classified as ASA 2, and patients with comorbidities that were not under control or limited daily activities were classified as ASA 3 [11].

Visual Analog Scale (VAS): VAS was used to measure the level of pain felt by patients in the preoperative and postoperative period. VAS is a scale that grades the severity of pain from 0 (no pain) to 10 (most severe pain). Data were collected from each patient at three different times: preoperative period, postoperative day 90, and postoperative day 180.

### Data Analysis

The data obtained as a result of the research were transferred to the computer environment and organized with Microsoft Excel package program and then analyzed with SPSS (Statistical Package for Social Sciences) 29.0 package program. Before starting the analysis, the suitability of the numerical data for normal distribution was examined with Skewness and Kurtosis tests. As a result of the analyses, it was concluded that the data came from a normal distribution. While categorical data are shown with frequency and percentage values, numerical data are shown with mean and standard deviation values since they meet the normality assumption. A Repeated Measure ANOVA Test was used since there were 3 repeated measurements in the study. In the evaluations made according to ASA, the Mann–Whitney U Test, which is a nonparametric test, was utilized since the number of people in the groups was 10 and 12. The statistical significance level was accepted as *p* < 0.05 for all tests. Pearson’s correlation analysis was conducted to determine the strength and direction of the relationships between the variables.

## 3. Results

In the patients who underwent a total knee arthroplasty, notable improvements were observed in the postoperative 30STS and TUG Tests compared to the preoperative values. Additionally, the pain levels, measured using the VAS, significantly decreased in the postoperative period. Descriptive statistics for these measurements, as well as comparisons between repeated assessments, performance level data, and analyses based on ASA scores, are presented in Table 1, Table 2, Table 3 and Table 4. These tables provide a comprehensive overview of the relationship between the preoperative functional test results and postoperative recovery.

The Skewness and Kurtosis values show whether the data are normally distributed or not. Tabachnick and Fidell state that the data are normally distributed when the Skewness and Kurtosis values are between −1.5 and +1.5. In this case, only the Day 90–Day 180 difference for the TUG score is not normally distributed. As shown in Table 1, the preoperative 30STS score was 8.16 ± 2.28, which increased to 13.70 ± 2.85 on day 90 and further to 15.35 ± 2.81 on day 180. Similar improvements were observed in the TUG and VAS scores, indicating significant functional recovery postoperatively.

**Figure 1:**
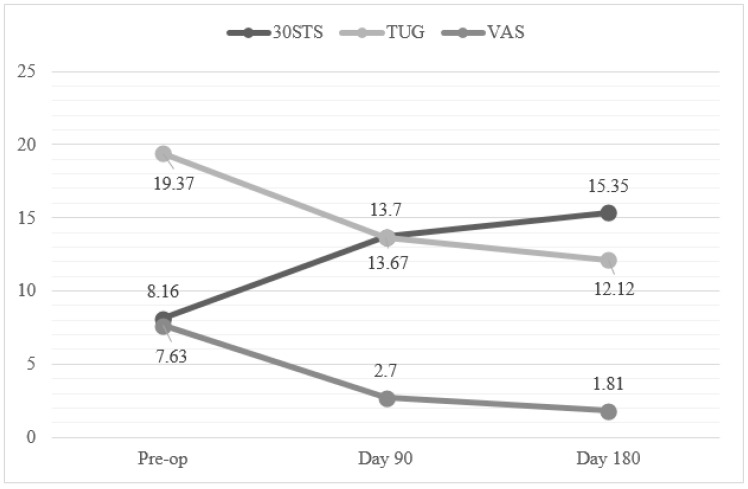
Mean values of 30STS and TUG Tests and VAS preoperatively and at day 90 and day 180.

For the 30STS score, the difference between the pre-op, day 90, and day 180 measurements is statistically significant (*p* < 0.001). The pre-op mean was 8.16 ± 2.28, the day 90 mean was 13.70 ± 2.85, and the day 180 mean was 15.35 ± 2.81 (Figure 1). As a result of the post hoc analysis, the difference between the pre-op and day 90 and day 180 measurements was found to be significant. After the pre-op value, an increase was observed on day 90 and day 180. The effect size was found to be high (η^2^ = 0.897).

The difference between the pre-op, day 90, and day 180 measurements for the TUG score was statistically significant (*p* < 0.001). The pre-op mean was 19.37 ± 8.89, the day 90 mean was 13.67 ± 5.52, and the day 180 mean was 12.12 ± 4.88. As a result of the post hoc analysis, the difference between the pre-op and day 90 and day 180 measurements was found to be significant. A decrease was observed on day 90 and day 180 after the pre-op value. The effect size was found to be high (η^2^ = 0.721).

The difference between the pre-op, day 90, and day 180 measurements for the VAS was statistically significant (*p* < 0.001). The pre-op mean was 7.63 ± 1.16, the day 90 mean was 2.70 ± 0.80, and the day 180 mean was 1.81 ± 0.63. As a result of the post hoc analysis, the difference between the pre-op and day 90 and day 180 measurements was found to be significant. A decrease was observed on day 90 and day 180 after the pre-op value. The effect size was found to be high (η^2^ = 0.953).

Table 2 indicates that both the functional performance (30STS and TUG scores) and pain levels (VAS scores) significantly improved in the postoperative period, with the most pronounced changes occurring within the first 90 days. These findings highlight the value of these tests in patient assessment and follow-up, particularly during the preoperative and early postoperative phases.

When the 30STS values were analyzed, while 93.0% of the patients had low results in the pre-op measurement, the rate of low results decreased to 25.6% on day 90, and the rate of intermediate results increased to 65.1%. At the end of the 180th day, the low rate was 7.0%, the medium rate was 58.1%, and the high rate was 34.9%.

When the TUG values were analyzed, while the pre-op measurement was high in 95.3%, high scores in 83.7% and medium scores in 16.3% were obtained at 90 days. At the end of the 180th day, the high rate decreased to 62.8%, the medium rate increased to 27.9%, and the low rate was 9.3%.

Table 3 demonstrates that both the 30STS and TUG performance levels improved significantly over time, with a notable shift from the low to the medium and high performance categories, particularly within the first 90 days.

There was no statistically significant difference between the pre-operative 30STS scores (*p* = 0.231) and TUG scores (*p* = 0.785) of the patients with ASA scores 2 and 3.

When the pre-op value for the 30STS score on day 90 was subtracted and the significance of the difference between the ASA 2 and ASA 3 patients was analyzed, the *p*-value was <0.001. In this case, there is a statistically significant difference between the ASA 2 and ASA 3 patients. While the improvement in the ASA 2 patients showed an average increase of 7.04 on the 90th day, this value was 3.44 in the ASA 3 patients. The improvement in the ASA 2 patients was significantly higher than that in the ASA 3 patients. Similarly, the change between pre-op and day 180 was significantly different in the patients with ASA scores of 2 and 3 (*p* < 0.05). At the end of the 180th day, the mean increase was 8.44 in the ASA 2 patients and 5.44 in the ASA 3 patients. There was no significant difference between day 90 and day 180 (*p* > 0.05).

When the significance of the difference between the ASA 2 and ASA 3 patients was analyzed by subtracting the pre-op value on the 90th day from the TUG score, the *p*-value was <0.001. When the measurement differences of the other TUG values and VAS values and the differences between the ASA 2 and ASA 3 patients were analyzed, no significant differences were observed (*p* > 0.05).

Table 4 demonstrates that the patients with ASA 2 scores showed significantly greater improvements in the 30STS and TUG scores compared to those with ASA 3 scores, particularly within the first 90 days postoperatively. These findings suggest that the ASA classification may be an important indicator of postoperative functional recovery, with ASA 2 patients exhibiting a more favorable rehabilitation trajectory.

For the 30STS score, TUG score, and VAS values, the correlations between the measurements were also analyzed. As a result of the correlation test, moderate positive correlations were found between the 30STS pre-op value and day 90 (r = 0.677, *p* < 0.001) and day 180 (r = 0.705, *p* < 0.001) measurements. The day 90 and day 180 values of the individuals with high pre-op values were also found to be high.

Highly positive relationships were found between the TUG score pre-op value and day 90 (r = 0.907, *p* < 0.001) and day 180 (r = 0.872, *p* < 0.001) measurements. The day 90 and day 180 values of the individuals with high pre-op values were also found to be high.

Moderate positive relationships were found between the VAS pre-op value and day 90 (r = 0.633, *p* < 0.001) and day 180 (r = 0.533, *p* < 0.001). The day 90 and day 180 values of the individuals with high pre-op values were also found to be high.

These findings suggest that the preoperatively administered tests applied may not fully overlap with the ASA score and that there was a significant improvement in the functional performance of the patients, especially in the first 90 days; moreover, this improvement was seen more clearly in ASA 2 patients.

## 4. Discussion

In this study, we evaluated the functional performance of patients undergoing TKA with the 30STS and TUG Tests and examined the relationship of these results with ASA scores and VAS values. Our findings revealed that both tests showed significant improvements in functional performance in the postoperative period.

Anesthesiologists usually assess the functional capacity of patients through anamnesis. However, for a more objective assessment, tests such as the Cardiopulmonary Exercise Test (CPET), 6-minute walk test (6MWT), and Five Repetition Sit and Stand Test (5STS) can be used. Silvapulle et al. stated that the CPET has high accuracy, while the 6MWT and the Incremental Speed Walking Test (ISWT) may be limited but useful for risk prediction [16]. According to a review study conducted by Argillander et al., the CPET has been shown to have a significantly better ability to predict postoperative outcomes compared to the 6MWT and ISWT, while the TUG Test has demonstrated inconsistencies in this regard [17]. Makker et al. emphasized that the 6MWT and 5STS may be useful tools in preoperative evaluation because they are low-cost and easily applicable, but it is difficult to replace the CPET because it is more comprehensive and provides a higher accuracy [18]. McCarthy et al. have proposed that the 30STS is a better measure of lower extremity endurance compared to the 5STS [19]. However, there are insufficient studies in the literature evaluating the relationship between the 30STS test and ASA scores. In our study, it was found that the results of the 30STS and TUG Tests did not show a significant correlation with the patients’ ASA scores.

Shulman et al. showed that patient-centered assessment tools may have a stronger capacity to predict disability-free survival (DFS) after surgery compared to the CPET. In their study, they found that patients who walked less than 435 m on the preoperative 6MWT had higher ASA scores, more chronic health problems and lower DFS rates. These findings suggest that the use of patient-centered tools such as the 6MWT and Duke Activity Status Index (DASI) instead of the CPET in preoperative risk assessments may be more effective and may play an important role in predicting long-term patient-centered outcomes [12]. In the study conducted by Staartjes et al., it was found that patients classified as ASA 3 had significantly longer TUG Test times [20]. However, to date, there have been no studies evaluating the relationship between the 30STS with ASA scores. In our study, we found that patient-centered 30STS and TUG Tests were effective in evaluating post-op functional performance and that this evaluation was related to the ASA scores.

Anesthesiologists use the ASA score when evaluating patients to predict and prevent postoperative complications. In the literature, there are studies examining the effect of the preoperative functional status and ASA score on the postoperative recovery process. For example, Shulman et al. found a significant correlation between the ASA score and the World Health Organization Disability Rating Scale 2.0 (WHODAS) and showed that patients with higher ASA scores had a higher risk of disability in the postoperative period [21]. In a study conducted by Dwyer et al. on patients who underwent total hip arthroplasty, it was found that the hospitalization period of patients with ASA 3 scores was longer than those with ASA 1 and 2 scores [22]. The effect of the preoperative physical status on the postoperative functional recovery was also emphasized in the study by Jonsson et al. It was found that the recovery process was accelerated in patients with a better preoperative functional level determined by a high New Mobility Score and a low ASA score [23]. Teni et al. showed that a low ASA score may positively affect not only the risk of surgical complications but also long-term health outcomes and quality of life [10]. Chen et al. showed that lower ASA scores resulted in better physical recovery and quality of life outcomes after hip fracture surgery in elderly patients [9]. In our study, the significant improvement in the 30STS and TUG scores of the ASA 2 group, especially in the first 90 days, is consistent with this finding and supports that the ASA score is an important indicator in predicting postoperative functional recovery.

In the literature, the preoperative functional performance has been shown to have significant effects on the postoperative recovery and complication risk. Smith et al. found that patients who walked more than 308 m on the 6MWT recovered better [24]. Ramos et al. stated that each 100 m decrease in the 6MWT distance increased the risk of complications by 30% [25]. Kristensen et al. also found that patients who completed the TUG Test in more than 20 s had a higher one-year mortality rate [26]. In a study by Taniguchi et al. in patients undergoing TKA, the preoperative TUG and STS tests were significantly associated with postoperative recovery. It was found that patients with a lower preoperative TUG duration exhibited better gait function postoperatively and that these tests were effective in predicting postoperative functional outcomes [27]. Similarly, Elings et al. reported that patients with lower ASA scores and shorter TUG times had shorter hospital stays and recovered faster in the postoperative period [28]. The correlation between the pre-op 30STS and TUG Test results and post-op 30STS and TUG Test results in our study emphasizes the importance of these easily applicable tests in patient follow-up.

Finally, it is thought that improvements in functional performance may be associated with TKA’s ability to reduce pain and increase joint range of motion. In the study conducted by Gautschi et al. on patients undergoing lumbar spine surgery, a significant relationship was observed between the TUG Test and Visual Analog Scale (VAS) scores in the preoperative and postoperative periods, and this relationship was reported to strengthen further after surgery [29]. Similarly, in our study, significant reductions in the VAS scores were recorded in the postoperative period, which paralleled the improvements in the functional test scores.

In the literature, multidisciplinary methods that can be used jointly by many branches in patient evaluation are gaining more and more attention. Our study revealed that the 30STS and TUG Tests were compatible with the ASA scores of the patients and that these tests can be an important tool in preoperative and postoperative evaluation. With a multidisciplinary team approach, healthcare professionals from different disciplines, from physiotherapists to anesthesiologists and surgeons, can improve patient care by using these tests as a common assessment tool.

Our study has some limitations. The limited sample size may restrict the generalizability of the results. Additionally, the retrospective design makes it challenging to establish causal relationships. The effects of demographic factors (age, gender, and socioeconomic status) and comorbidities on functional outcomes were not evaluated. Future prospective studies with larger sample sizes considering these factors will contribute to validating the results and enhancing their generalizability.

## 5. Conclusions

This study demonstrates that the preoperative results of the 30STS and TUG Tests correlate with postoperative outcomes, indicating that these tests are effective and practical tools for predicting and evaluating functional performance in patients undergoing TKA during the postoperative period. Notably, within the ASA 2 group, significant improvements observed during the early period (first 90 days) underscore the ASA score as a crucial indicator for predicting postoperative recovery. However, the lack of a significant difference between the preoperative test values and ASA scores suggests that these tests may not be sufficiently reliable for objectively assessing the functional capacity within ASA scoring. The better preoperative test outcomes in the ASA 2 patients suggest that integrating these tests with anesthetic evaluations could be beneficial in predicting the postoperative functionality. We believe that employing these and similar tests will contribute to a more comprehensive patient assessment, thus supporting a multidisciplinary approach to care. These findings need to be validated in clinical practice and examined for reproducibility across different patient populations. Future studies should comprehensively evaluate the applicability of these tests in various patient groups, their impact on long-term outcomes, and their compatibility with different surgical techniques.

## Figures and Tables

**Table 1:** The statistics of 30STS score, TUG score, and VAS values for pre-op, 90th day, and 180th day measurements.

	$\bar{x}$ ± SD	Min–Max	Skewness	Kurtosis
**30STS Score**				
Pre-op	8.16 ± 2.28	4–14	0.208	−0.274
Day 90	13.70 ± 2.85	8–19	−0.398	−0.843
Day 180	15.35 ± 2.81	9–20	−0.484	−0.460
Day 90–Pre-op	5.53 ± 2.14	1–9	−0.287	−1.076
Day 180–Pre-op	7.19 ± 2.08	3–11	−0.210	−0.750
Day 180–Day 90	1.70 ± 0.94	0–4	0.837	0.791
**TUG Score**				
Pre-op	19.37 ± 8.89	9–43	1.122	0.225
Day 90	13.67 ± 5.52	8–30	1.275	0.837
Day 180	12.12 ± 4.88	7–26	1.207	0.529
Pre-op–Day 90	0.77 ± 0.53	0–2	−0.224	−0.076
Pre-op–Day 180	1.21 ± 0.56	0–2	0.053	−0.084
Day 90–Day 180	0.49 ± 0.51	0–1	0.048	−2.098
**VAS**				
Pre-op	7.63 ± 1.16	4–9	−1.154	1.657
Day 90	2.70 ± 0.80	1–4	0.036	−0.536
Day 180	1.81 ± 0.63	1–3	0.151	−0.449
Pre-op–Day 90	4.93 ± 1.01	3–7	−0.146	−0.261
Pre-op–Day 180	5.81 ± 1.18	3–8	−0.259	−0.183
Day 90–Day 180	0.88 ± 0.76	0–2	0.202	−1.221

$\bar{x\;}$ ± SD; mean ± standard deviation, CA (α); Cronbach’s Alpha, 30STS, 30-Second Sit-to-Stand Test; TUG, Timed Up-and-Go Test; VAS, Visual Analog Scale; $\bar{x}$ ± SD, mean ± standard deviation; Min–Max, minimum–maximum values.

**Table 2:** The results of the analysis of the intra-group comparisons of 30STS score, TUG score, and VAS values.

	$\bar{x}$ ± SD	F	*p*	Partial Eta Squared
**30STS Score**				
Pre-op	8.16 ± 2.28 ^a^	366,395	<0.001	0.897
Day 90	13.70 ± 2.85 ^b^			
Day 180	15.35 ± 2.81 ^c^			
**TUG Score**				
Pre-op	19.37 ± 8.89 ^a^	78,581	<0.001	0.721
Day 90	13.67 ± 5.52 ^b^			
Day 180	12.12 ± 4.88 ^c^			
**VAS**				
Pre-op	7.63 ± 1.16 ^a^	845,967	<0.001	0.953
Day 90	2.70 ± 0.80 ^b^			
Day 180	1.81 ± 0.63 ^c^			

Repeated Measure ANOVA Test, *p* < 0.001. The letters ^a,b,c^ indicate the results of post hoc analysis. The difference between different letters is significant. 30STS, 30-Second Sit-to-Stand Test; TUG, Timed Up-and-Go Test; VAS, Visual Analog Scale; $\bar{x}$ ± SD, mean ± standard deviation; F, F-statistic value; *p*, *p*-value; Partial Eta Squared, effect size.

**Table 3:** The distribution of performance levels of 30STS and TUG Tests preoperation and at day 90 and day 180.

	Pre-op		Day 90		Day 180	
	N	%	N	%	N	**%**
**30STS Performance Level**						
Low	40	93.0%	11	25.6%	3	7.0%
Medium	3	7.0%	28	65.1%	25	58.1%
High	0	0.0%	4	9.3%	15	34.9%
**TUG Performance Level**						
Low	0	0.0%	0	0.0%	4	9.3%
Medium	2	4.7%	7	16.3%	12	27.9%
High	41	95.3%	36	83.7%	27	62.8%

30STS, 30-Second Sit-to-Stand Test; TUG, Timed Up-and-Go Test; N, number of patients; %, percentage of patients.

**Table 4:** The comparison of changes in 30STS, TUG, and VAS values according to ASA scores.

	ASA	n	Mean	SD	Median	Min	Max	U	*p*
**30STS Score**									
Day 90–Pre-op	2	25	7.04	1.17	7	4	9	5.360	<0.001
	3	18	3.44	1.20	4	1	6		
Day 180–Pre-op	2	25	8.44	1.39	8	5	11	4.622	<0.001
	3	18	5.44	1.58	5	3	9		
Day 180- Day 90	2	25	1.48	0.82	1	0	4	1.575	0.115
	3	18	2.00	1.03	2	1	4		
**TUG Score**									
Pre-op–Day 90	2	25	0.96	0.45	1	0	2	2.838	0.005
	3	18	0.50	0.51	0.5	0	1		
Pre-op–Day 180	2	25	1.32	0.69	1	0	2	1.792	0.073
	3	18	1.06	0.24	1	1	2		
Day 90–Day 180	2	25	0.44	0.51	0	0	1	0.739	0.460
	3	18	0.56	0.51	1	0	1		
**VAS**									
Pre-op–Day 90	2	25	4.88	0.88	5	3	6	0.285	0.776
	3	18	5.00	1.19	5	3	7		
Pre-op–Day 180	2	25	5.80	1.08	6	4	8	0.129	0.898
	3	18	5.83	1.34	6	3	8		
Day 90–Day 180	2	25	0.92	0.76	1	0	2	0.382	0.702
	3	18	0.83	0.79	1	0	2		

ASA, American Society of Anesthesiologists; 30STS, 30-Second Sit-to-Stand Test; TUG, Timed Up-and-Go Test; VAS, Visual Analog Scale; n, number of patients; SD, standard deviation; U, Mann–Whitney U Test Statistic; *p*, *p*-value.

## Data Availability

The raw data supporting the results of this study are available upon reasonable request from the corresponding author. Due to hospital policies and ethical restrictions, the data can only be shared in anonymized form. To ensure compliance with institutional regulations, a data transfer agreement may be required prior to sharing the data.

## Abbreviations

The following abbreviations are used in this manuscript:

TKA	Total knee arthroplasty
30STS	30-Second Sit-to-Stand Test
TUG	Timed Up-and-Go
LD	American Society of Anesthesiologists
VAS	Visual Analog Scale
CPET	CPET
6MWT	6-minute walk test
5STS	Five Repetition Sit and Stand Test
ISWT	Incremental Speed Walking Test

## Appendix A

**Table A1:** Table showing the mean values of the 30STS Test and TUG Test according to age and gender [14,15].

Tests	30STS		TUG	
Age Group (Years)	Women—Average	Men—Average	Women—Average	Men—Average
60–64	12–17	14–19	7–9 s	7–8 s
65–69	11–16	12–18		
70–74	10–15	12–17	8–10 s	7–11 s
75–79	10–15	11–17		
80–84	9–14	10–15	9–12 s	9–11 s
85–89	8–13	8–14		
90–94	4–11	7–12		
